# Integrated transcriptome and metabolome analysis reveals anthocyanin biosynthesis mechanisms in pepper (*Capsicum annuum* L.) leaves under continuous blue light irradiation

**DOI:** 10.1186/s12870-024-04888-x

**Published:** 2024-03-23

**Authors:** Yao Zhou, Weisheng Wu, Ying Sun, Yiyu Shen, Lianzhen Mao, Yunhua Dai, Bozhi Yang, Zhoubin Liu

**Affiliations:** Engineering Research Center of Education Ministry for Germplasm Innovation and Breeding New Varieties of Horticultural Crops, Key Laboratory of Vegetable Biology of Hunan Province, College of Horticulture, Hunan Agricultural University, Changsha, 410128 Hunan China

**Keywords:** Chili pepper, Continuous photoperiod, Anthocyanin biosynthesis, Blue light

## Abstract

**Background:**

Different metabolic compounds give pepper leaves and fruits their diverse colors. Anthocyanin accumulation is the main cause of the purple color of pepper leaves. The light environment is a critical factor affecting anthocyanin biosynthesis. It is essential that we understand how to use light to regulate anthocyanin biosynthesis in plants.

**Result:**

Pepper leaves were significantly blue–purple only in continuous blue light or white light (with a blue light component) irradiation treatments, and the anthocyanin content of pepper leaves increased significantly after continuous blue light irradiation. This green-to-purple phenotype change in pepper leaves was due to the expression of different genes. We found that the anthocyanin synthesis precursor-related genes *PAL* and *4CL*, as well as the structural genes *F3H*, *DFR*, *ANS*, *BZ1*, and *F3’5’H* in the anthocyanin synthesis pathway, had high expression under continuous blue light irradiation. Similarly, the expression of transcription factors *MYB1R1-like, MYB48, MYB4-like isoform X1, bHLH143-like, and bHLH92-like isoform X3*, and circadian rhythm-related genes *LHY and COP1*, were significantly increased after continuous blue light irradiation. A correlation network analysis revealed that these transcription factors and circadian rhythm-related genes were positively correlated with structural genes in the anthocyanin synthesis pathway. Metabolomic analysis showed that delphinidin-3-O-glucoside and delphinidin-3-O-rutinoside were significantly higher under continuous blue light irradiation relative to other light treatments. We selected 12 genes involved in anthocyanin synthesis in pepper leaves for qRT-PCR analysis, and the accuracy of the RNA-seq results was confirmed.

**Conclusions:**

In this study, we found that blue light and 24-hour irradiation together induced the expression of key genes and the accumulation of metabolites in the anthocyanin synthesis pathway, thus promoting anthocyanin biosynthesis in pepper leaves. These results provide a basis for future study of the mechanisms of light quality and photoperiod in anthocyanin synthesis and metabolism, and our study may serve as a valuable reference for screening light ratios that regulate anthocyanin biosynthesis in plants.

**Supplementary Information:**

The online version contains supplementary material available at 10.1186/s12870-024-04888-x.

## Background

Pepper (Capsicum annuum L.) is an important vegetable crop in China because of its high nutritional value and wide agronomic range [1]. Due to differences in metabolites, pepper leaves and fruits occur in various colors, including green, yellow, and purple. Anthocyanins are an important secondary flavonoid metabolite in plants, and anthocyanins usually produce red, blue, or purple colors in leaves and fruits (if cell fluid is acidic, red is produced, and if cell fluid is alkaline, blue is produced) [2]. Anthocyanins have pollination, seed dispersal, and resistance functions for plants [3, 4], and they also have health benefits for humans in the fields of anti-aging, beauty, and cardiovascular disease [5, 6]. The biosynthesis of plant anthocyanins is affected by many external environmental factors, such as light, hormones, sugars, and temperature, which can promote or inhibit anthocyanin synthesis [7]. Among these factors, light is considered to be key: light intensity, light quality, and photoperiod affect anthocyanin biosynthesis [8].

Previous studies have shown that anthocyanin glycoside-related genes are downregulated under low light or darkness in fruits and leaves, resulting in a reduction in anthocyanin content [9]. For example, compared to shaded pericarps, apple fruits exposed to sunshine collected more anthocyanin glycosides [10]. Light quality is crucial in anthocyanin synthesis and accumulation [11]. The effects of light quality on anthocyanin biosynthesis and accumulation vary with plant species, as do the regulatory effects of light quality on genes related to anthocyanin synthesis [12]. In a study of bayberry fruits, blue light induced the expression of genes related to anthocyanin synthesis, increasing the accumulation of anthocyanins in fruits [13]. Tao et al. [14] found that blue light strongly induced the accumulation of anthocyanins in red pear skin, while red light had almost no effect. Adding UV-A and Blue light irradiation to the growth environment of lettuce increased anthocyanin concentration in young lettuce leaves by 11% and 31%, respectively [11]. Blue light increased anthocyanin content and transcript levels of anthocyanin biosynthesis genes more than white or red light in strawberries [15]. Zhang et al. [16] confirmed that blue light significantly increased the anthocyanin content of Cabernet Sauvignon grapes compared to white light.

Studies of photoperiodic regulation suggest that the circadian clock is fundamental (in addition to photoreceptors) in detecting and responding to photoperiodic changes [8]. Therefore, plant anthocyanin biosynthesis is strictly regulated by circadian rhythms, forming a complex secondary metabolic network [17]. Continuous illumination is an important tool to understand the circadian rhythms of plants [18]. Continuous light (CL) refers to an environment where plants grow under continuous 24-hour light [19]. Photoperiod directly affects the synthesis of plant secondary metabolites [20–22]. Several studies have shown that prolonged illumination will increase the accumulation of anthocyanins in plants. Carvalho et al. [22] found an increase in gene expression related to the flavonoid synthesis pathway in sweet potato leaves after 30 d of treatment with 16 h of sunlight compared to 8 h of sunlight; this led to increased anthocyanin and flavonoid content. Similarly, a longer light duration can significantly increase the concentration of nutrients such as anthocyanins and phenolic compounds in cucumber seedlings, thus improving their nutritional quality [23]. Studies have also confirmed that continuous light has a positive effect on the growth, development, yield, and quality of horticultural plants [24–26].

Transcription factors play an important role in plant growth and development, secondary metabolic regulation by regulating gene expression [27, 28]. Transcriptional regulation modifies gene expression by changing the transcription rate, thereby affecting the biosynthesis of anthocyanins in plants [29]. Studies have shown that in the anthocyanin synthesis pathway of Arabidopsis thaliana, MYB transcription factor genes interact with bHLH transcription factor genes and WD40-repeat transcription factor genes to form the MYB-bHLH-WD40 protein complex (MBW) [30]. The R2R3-MYB plays a central role in the regulation of anthocyanin biosynthesis and is a key element in the MBW complex that governs anthocyanin synthesis [31]. At present, R2R3-MYB has been found in many horticultural crops, such as maize [32], grape [33], tomato [34], and apple [10], and participates in regulating anthocyanin biosynthesis in response to light induction. In Solanaceae, the bHLH branch involved in the regulation of plant anthocyanin biosynthesis includes mainly the orthologous genes PhAN1 and PhjAF13 in petunia. Related studies have shown that compared with uncolored peppers and eggplant, the PhAN1 homologous genes CabHLH and SmbHLH were highly expressed in colored fruits and were positively correlated with anthocyanin structural genes and accumulation levels [35–37]. D’Amelia et al. confirmed that MYB transcription factors can interact with StAN1 and StbHLH [38]. In the WD40 protein family, TTG1 is an important transcription factor related to anthocyanin synthesis. The WD40 protein can provide a stable platform for MYB and bHLH proteins to form a MBW complex [39]. In addition to MBW, other transcription factor families have also been shown to play a role in flavonoid biosynthesis in higher plants, such as bZIP, NAC, and WRKY [31]. Nowadays, more and more consumers prefer foods rich in anthocyanins; thus, efficiently regulating the biosynthesis of anthocyanins in plants is of great significance. We studied the effects of monochromatic blue light and monochromatic red light on anthocyanin synthesis in pepper leaves under continuous light conditions. The pepper leaf transcriptome and metabolome were sequenced to explore the genes affecting leaf color change relative to light quality and photoperiod. Our intent was to identify the functional role of key genes in anthocyanin metabolism and to identify the mechanism of molecular regulation of anthocyanins in response to light quality and photoperiod. These results will provide a foundation for further screening light ratios that will regulate anthocyanin synthesis and enhance the resistance of pepper seedlings.

## Results

### Phenotype identification and pigment content analysis

**Fig. 1:**
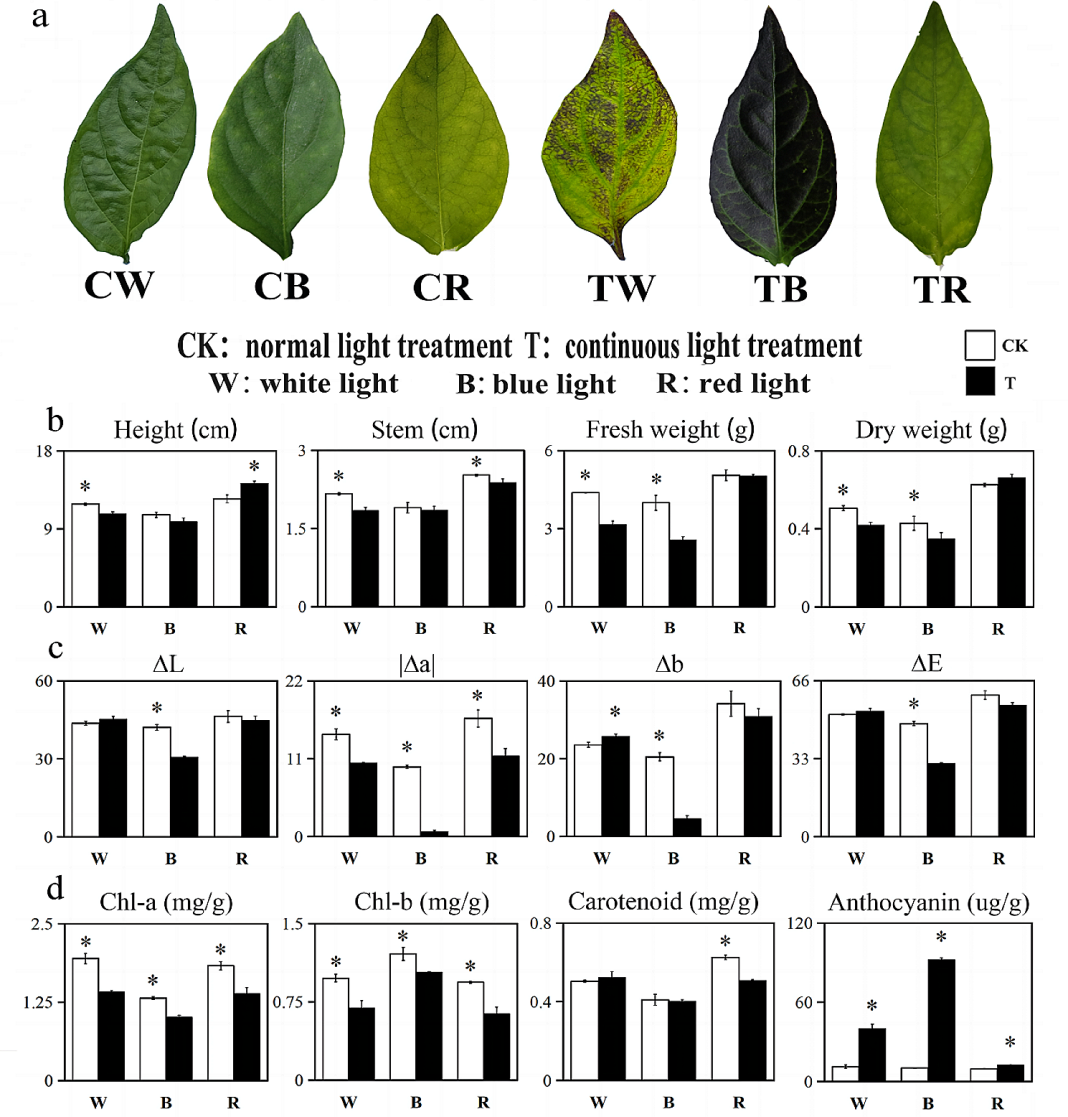
Phenotypic and leaf pigment content analysis of pepper under different light treatment groups.CK: normal light treatment(14 h light/10 h dark); T: continuous light treatment 24 h light/0 h dark. CW: normal white light treatment; CB: normal blue light treatment; normal red light treatment. TW: continuous white light treatment; TB: continuous blue light treatment; TR: continuous blue light treatment. (**a**) Phenotypic identification of pepper leaves. (**b**) Analysis of growth indicators of pepper plants; (**c**) Chroma values of pepper leaves. (**d**) Photosynthetic pigment content and anthocyanin content. *, *P-value* < 0.05

Pepper leaves under different light treatments had significant color differences (Fig. 1a). Our results showed that pepper leaves treated with continuous blue light were purple, and a small amount of purple was also distributed in peppers under constant white light. In contrast, leaves experienced a significant change to yellow after red light treatment. Pepper growth was remarkable after red light treatment but significantly inhibited after continuous blue light treatment (Fig. 1b). Colorimetric measurements showed that color luminance (ΔL), red (|Δa|), yellow (Δb), and total chromaticity (ΔE) values in pepper leaves after blue light treatment were considerably lower than those of pepper leaves in white or red light treatments, and the continuous blue light treatment (TB) colorimetric parameter was significantly lower than in the other light treatments (Fig. 1c). Chlorophyll a and b under a normal photoperiod were substantially higher than under a continuous photoperiod. However, the highest anthocyanin contents were found in TB (continuous blue light treatment), at 92 ug/g, and in TW (continuous white light treatment), at 40 ug/g (Fig. 1d). This was consistent with changes in leaf color after continuous light treatment, indicating that change in the light environment had great influence on leaf color.

### Metabolome analysis of pepper leaves after different light treatments

**Fig. 2:**
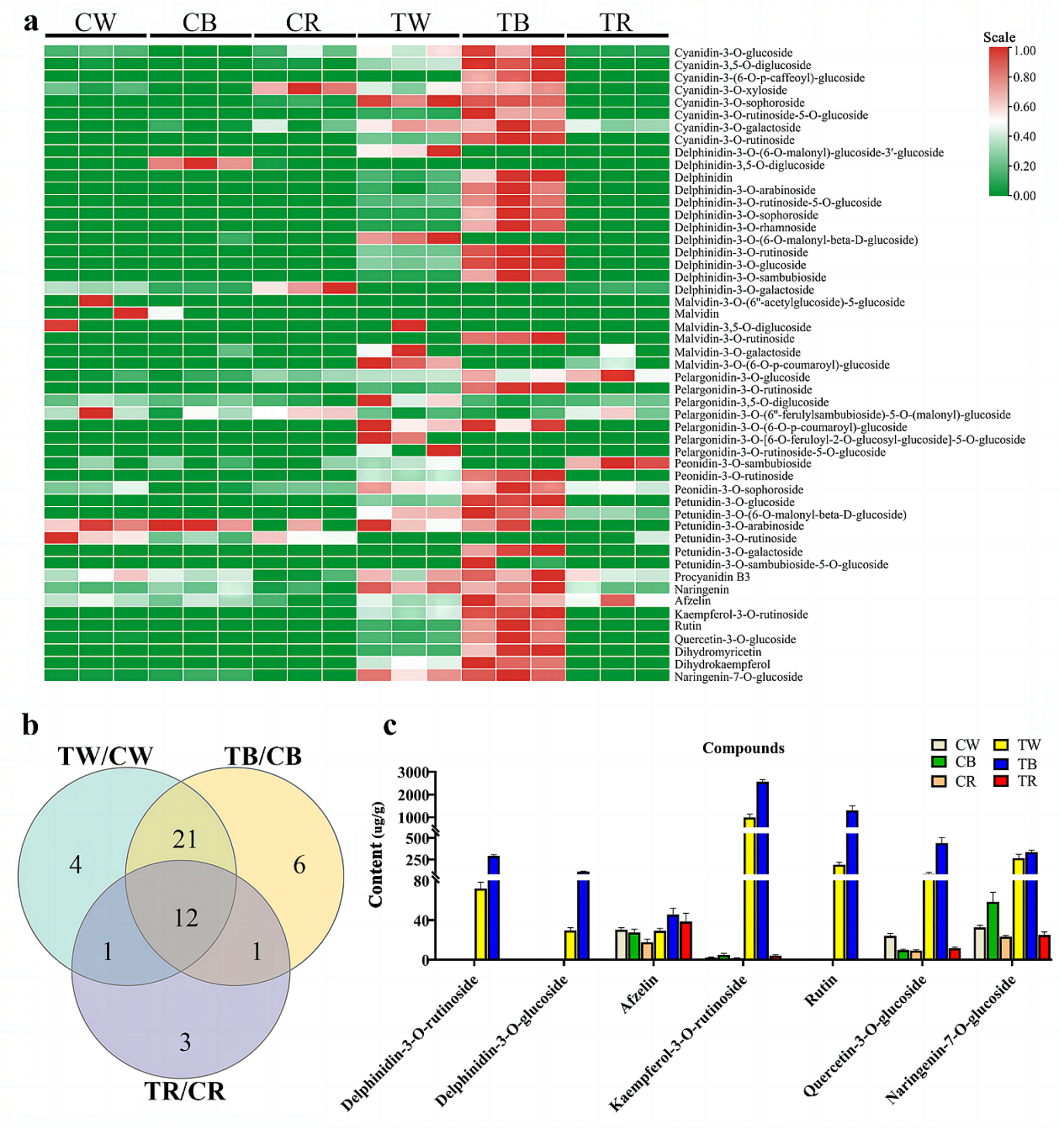
Metabolome analysis of pepper leaves under different light treatment groups. (**a**) Heat map of 51 anthocyanin contents. (**b**) Venn diagram between different light treatments under the same light quality. (**c**) histogram of differential metabolite contents

To better understand the process of leaf color change in pepper, liquid chromatography tandem mass spectrometry (LC-MS/MS) was used to analyze anthocyanin metabolites in pepper leaves in each treatment group, and 108 anthocyanin compounds were identified. (Table S1). By subjecting these compounds to unit variance scaling (UV) and hierarchical cluster analysis (HCA), we identified 12 delphinidin compounds, eight cyanidin compounds, eight flavonoids, seven pelargonidin compounds, six malvidin compounds, six petunidin compounds, three peonidin compounds, and one procyanidin compound (Fig. 2a, Table S2).

A total of 12 differential metabolites were compared between TW/CW, TB/CB, and TR/CR, with content values > 5 as the screening condition (Fig. 2b). The results showed significant variation in the levels of delphinidin-3-O-glucoside, delphinidin-3-O-rutinoside, fzelin, kaempferol-3-O-rutinoside, naringenin-7-O-glucoside, rutin, and quercetin-3-O-glucoside under different light conditions (Fig. 2c). The metabolic content in the TB treatment was notably higher than in other light treatments, suggesting that these compounds influence the formation of purple leaves.

### Transcriptome analysis of pepper leaves after different light treatments

**Fig. 3:**
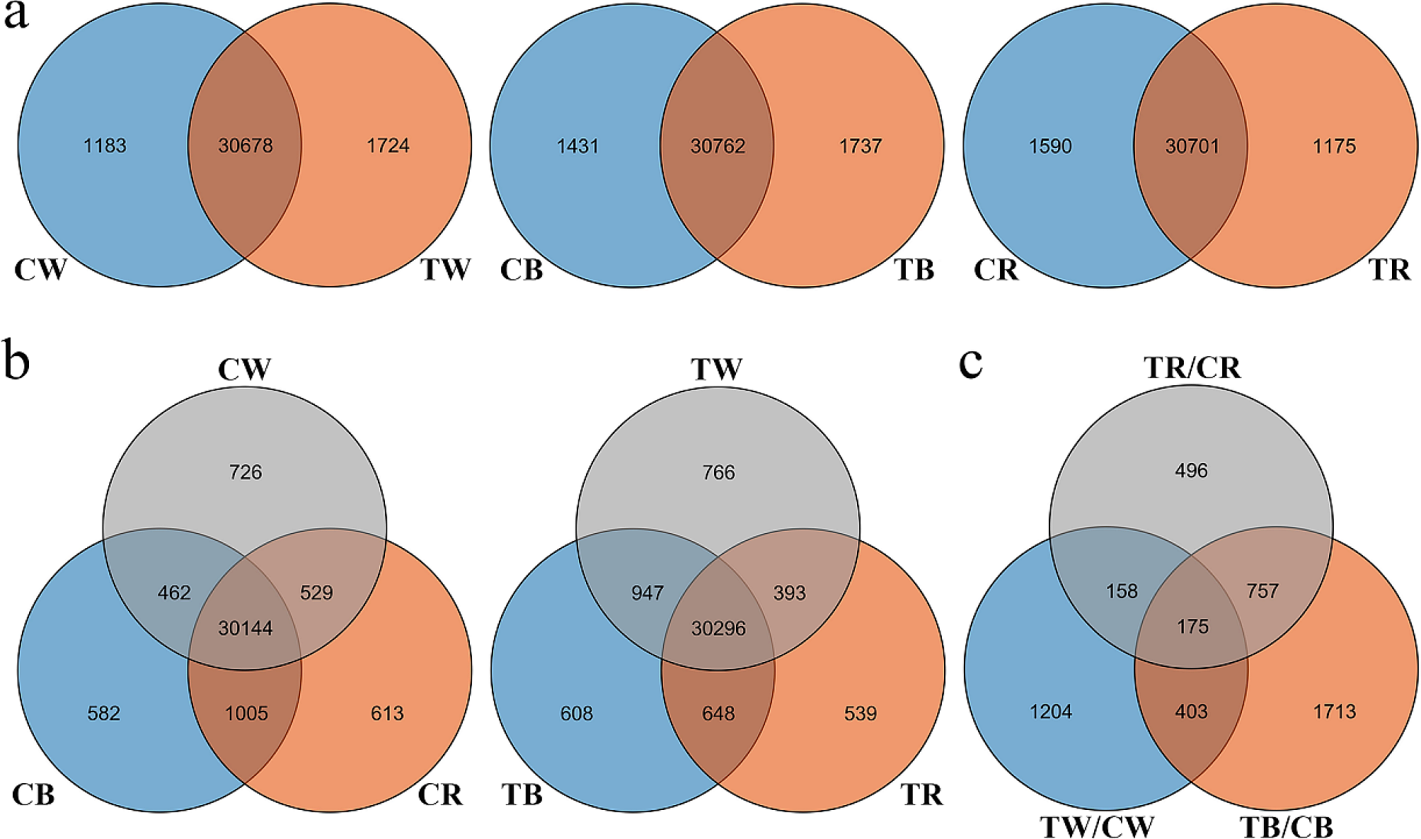
Statistical analysis of genes identified by transcriptome analysis. (**a**) Veen of quantified genes under normal light and continuous light conditions in the same light quality. (**b**) Veen of quantified genes under normal light and continuous light. (**c**) Venn of differentially expressed genes between different light treatments in the same light quality

Illumina NovaSeq6000 sequencing was performed on 18 samples. For each sample, 6.23Gb of clean data was generated, with a Q30 base percentage of 92.94% or greater. Subsequently, the data were summarized, and 527,312,454 clean reads were obtained. After removing low-quality reads and rRNA, the clean reads were compared to the Capsicum_annuum.Zunla-1_v2.0.genome.fa database (https://www.ncbi.nlm.nih.gov/genome/10896), with a comparison rate ranging from 90.49 to 95.89%. A total of 46,108 genes were identified in the sample, of which 35,318 genes were quantified (Table S3). A total of 30,296 and 30,144 genes were quantified in the continuous and normal photoperiodic treatments, respectively. Among them, 974 genes were quantified between TB and TW. Some genes were quantified only under a particular light treatment, with 766, 608, and 539 specific genes under the TW, TB, and TR treatments, respectively. Subsequently, the results of two different treatment conditions showed that the number of genes quantified by TW and TB was 1,724 and 1,737, respectively (Fig. 3a–b).

### Analysis of differentially expressed genes (DEGs)

By comparing gene expression in different light treatments, 16,243 DEGs were identified (Table S4. There were 4,544, 7,936, and 5,023 DEGs in TW/CW, TB/CB, and TR/CR, respectively, and there were 2,049, 4,290, and 2,758 upregulated genes and 2,494, 3,646, and 2,265 downregulated genes in TW/CW, TB/CB, and TR/CR, respectively (Table S5, Supplementary Fig. 1). There were 175 DEGs in each comparison group, while 1,204, 1,713 and 496 genes were significantly different only in TW/CW, TB/CB, and TR/CR, respectively (Fig. 3c). Further analysis showed that anthocyanidin synthesis precursor genes and chlorophyll synthesis genes were significantly different under each treatment. Among them, the expression of *PAL* (Capana05g002560) and *4CL* (Capana03g001365) genes under TB was 1.43-fold and 2.49-fold higher, respectively, than under CB. While chlorophyll synthesis-related genes *POR* (Capana00g004560) and *LIP* (Capana02g002261) were higher under CB, these genes exhibited a significant decrease in expression under TB, by 2.15-fold and 2.41-fold, respectively. The differential expression of genes related to anthocyanin synthesis precursors and chlorophyll synthesis may be an important reason for the apparent change in leaf color under continuous blue light treatment.

### Functional enrichment analysis for DEGs

**Fig. 4:**
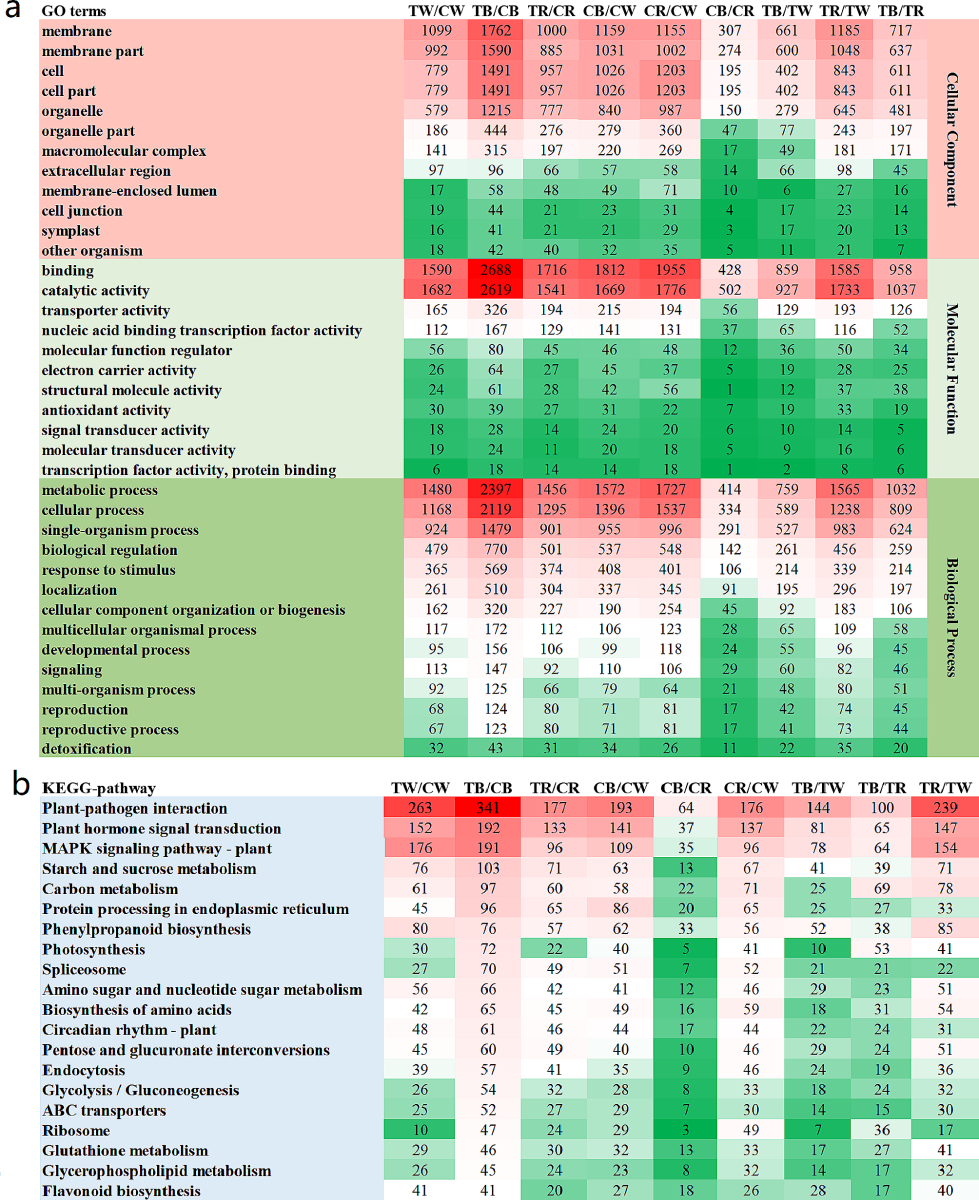
(**a**) GO analysis of differentially expressed genes. (**b**) KEGG analysis of differentially expressed genes

The classification of DEGs using the Gene Ontology (GO) database revealed that these DEGs were annotated to one or more GO terms covering three main branches: cellular components, molecular functions, and biological processes. These branches could be further classified into 18, 15, and 21 terms. Analysis of the top 37 GO terms with the largest number of DEGs (Fig. 4a) showed that these DEGs were mainly enriched in membrane, membrane part, cell, and organelle. Binding processes and catalytic activity were predominant in the molecular functional categories. In terms of biological processes, metabolic processes, and cellular processes, single-organism processes had the highest number of DEGs. We found that the degree of enrichment in CC, MF and BP function bars was markedly higher in the TB/CB group than in other light treatments. Abundant DEGs in these GO terms may be one of the reasons for anthocyanin synthesis and accumulation in pepper leaves under TB.

To gain a deeper understanding of the metabolic pathways that play an essential role in anthocyanin synthesis in pepper leaves, we selected the top 20 DEGs from the Kyoto Encyclopedia of Genes and Genomes (KEGG) pathways for the nine light comparison groups for enrichment analysis (Fig. 4b). The results showed that DEGs were significantly enriched in all light comparison groups, primarily in plant-pathogen interactions, plant-hormone signal transduction, and MAPK signaling pathway-plant. The number of DEGs in the TR/CR group was significantly less than in the TW/CW and TB/CB groups, suggesting that differential expression of genes in the above pathways may lead to leaf color differences under different light quality and photoperiod treatments. We also found that the phenylpropanoid biosynthesis pathway, flavonoid biosynthesis pathway, and circadian rhythm-plant pathway related to anthocyanin biosynthesis were significantly enriched in DEGs in the TB/CB and TW/CW groups, and the genes enriched in these three pathways were upregulated in the TB and TW groups. This suggests that differentially expressed genes in these metabolic pathways may be involved in forming purple colored pepper leaves.

### Gene expression patterns in the anthocyanin pathway

**Fig. 5:**
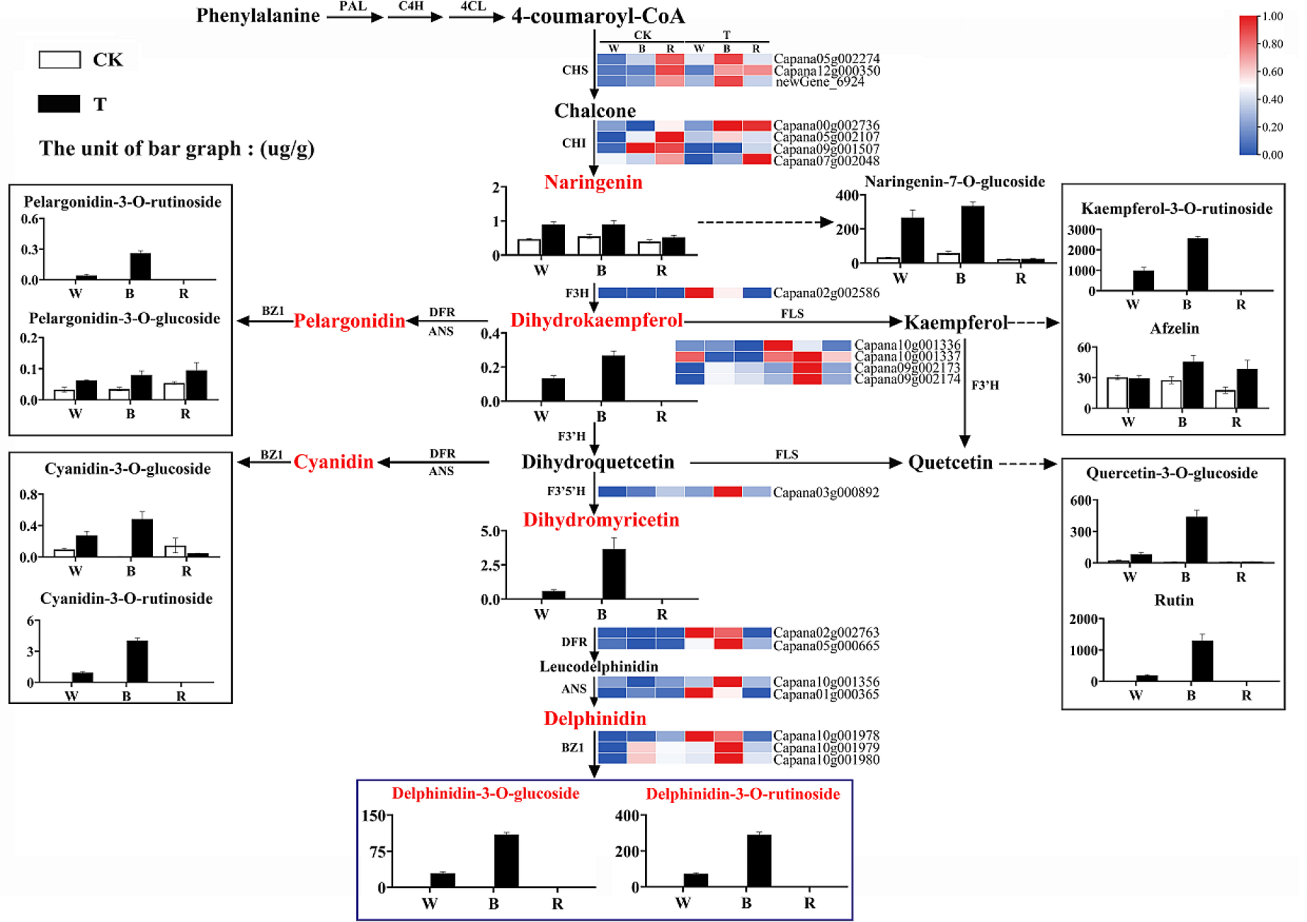
Expression of anthocyanin pathway genes and metabolites. W, white light; B, blue light; R, red light; CK: normal light treatment; T: continuous light treatment. Solid line: generation process; dashed line: generation of subsequent reactions; the red font: substances with content in the anthocyanin pathway; purple box, delphinium derivatives

To further investigate the effects of light quality and photoperiod on anthocyanin synthesis in pepper leaves, we analyzed the structural genes and anthocyanin metabolites involved in the anthocyanin synthesis pathway (Fig. 5). The expression of *CHS* (Capana05g002274, Capana12g000350, and newGene_6924) and *CHI* (Capana00g002736) in pepper leaves was significantly higher under TB treatment, which resulted in more intermediates, such as naringenin and naringenin-7-O-glucoside, being catalyzed via TB treatment. Meanwhile, the high expression of *F3H* (Capana02g002586) in the TW and TB treatments led to further catalytic formation of higher amounts of dihydrokaempferol. With dihydrokaempferol as an intermediate in the TW and TB groups, *FLS* (Capana09g002173 and Capana09g002174), *F3’5’H* (Capana03g000892), *DFR* (Capana05g000665), *ANS* (Capana10g001356), and *BZ1* (Capana10g001978, Capana10g001979, and Capana10g001980) were strongly expressed and further catalyzed the synthesis of large amounts of delphinidins. These results suggest that continuous blue and white light irradiation elevated the expression of relevant genes in the anthocyanin synthesis pathway, leading to significant accumulation of delphinidins, which were the main cause of purple color in leaves.

### Gene expression patterns in the circadian rhythm-plant pathway

**Fig. 6:**
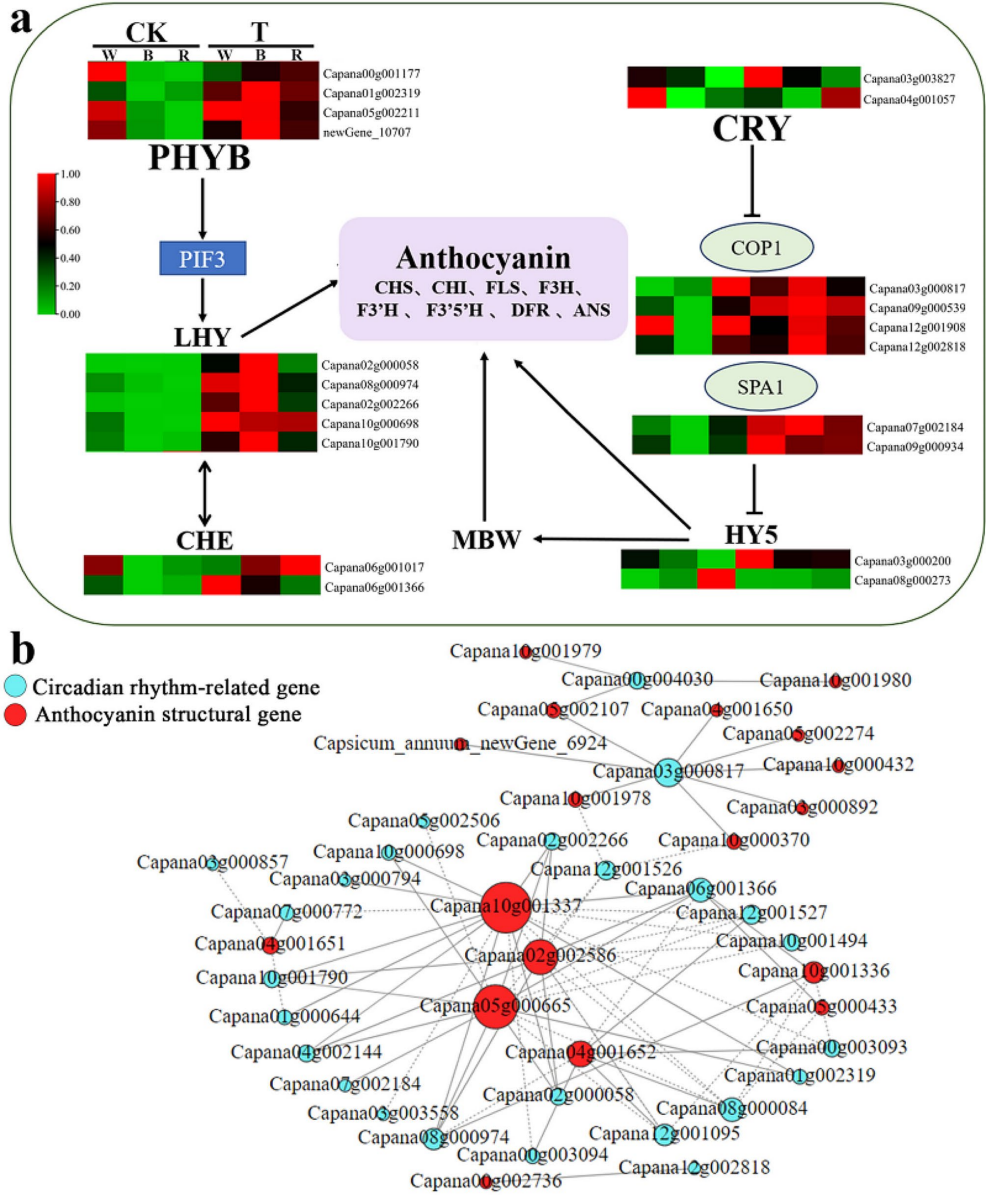
Combined analysis of differentially expressed circadian rhythm-plant genes and differentially expressed anthocyanin synthesis genes. (**a**) Analysis of differentially expressed genes in the circadian rhythm-plant pathway (**b**) Correlation network between circadian rhythm-related gene and anthocyanin structural gene. Only Pearson correlation coefficients (PCC) ≥ 0.80 or ≤ -0.80 are shown. solid line, positive correlation between genes; dashed line, negative correlation between genes.

The results of KEGG enrichment analysis showed that pepper leaves under continuous blue light had many DEGs in the circadian rhythm pathway, so we performed an in-depth analysis of genes related to this pathway (Fig. 6a). The expression of the red light receptor *PHYB* (Capana01g002319), upstream of the circadian pathway, was higher under the continuous blue light treatment and significantly higher than under the normal blue light treatment. In comparison, the expression of the blue light receptor *CRY* (Capana04g001057) was lower under the blue light treatment and significantly lower than under the other light treatments. Most genes showed significantly higher expression after continuous light treatment compared with normal light treatment, such as *LHY* (Capana02g002266, Capana02g00058, Capana10g000698, Capana10g001790, and Capana08g000974), *SPA1* (Capana17g002184), *CHE* (Capana06g001366), and *COP1* (Capana12g002818). Among them, the five *LHY* genes detected in pepper leaves were higher after TW, TB, and TR treatments than CW, CB, and CR treatments, with the highest expression after TB treatment and the lowest expression after CB treatment.

To investigate whether plant circadian rhythms played a role in anthocyanin biosynthesis, we analyzed correlations between anthocyanin pathway structural genes and circadian rhythm pathway genes (Fig. 6b). The results showed that *COP1* (Capana03g000817) was positively correlated with various anthocyanin structural genes, such as *CHS* (Capana05g002274), *CHI* (Capana05g002107), *F3’5’H* (Capana03g000892), and *BZ1* (Capana10g001978) and that *LHY* (Capana02g000058) was also positively correlated with *DFR* (Capana05g000665), FLS (Capana10g001337), and *F3H* (Capana02g002586). This suggests that light induced changes in the expression of structural genes in the anthocyanin biosynthetic pathway, leading to changes in anthocyanin content.

### Transcription factor analysis

**Fig. 7:**
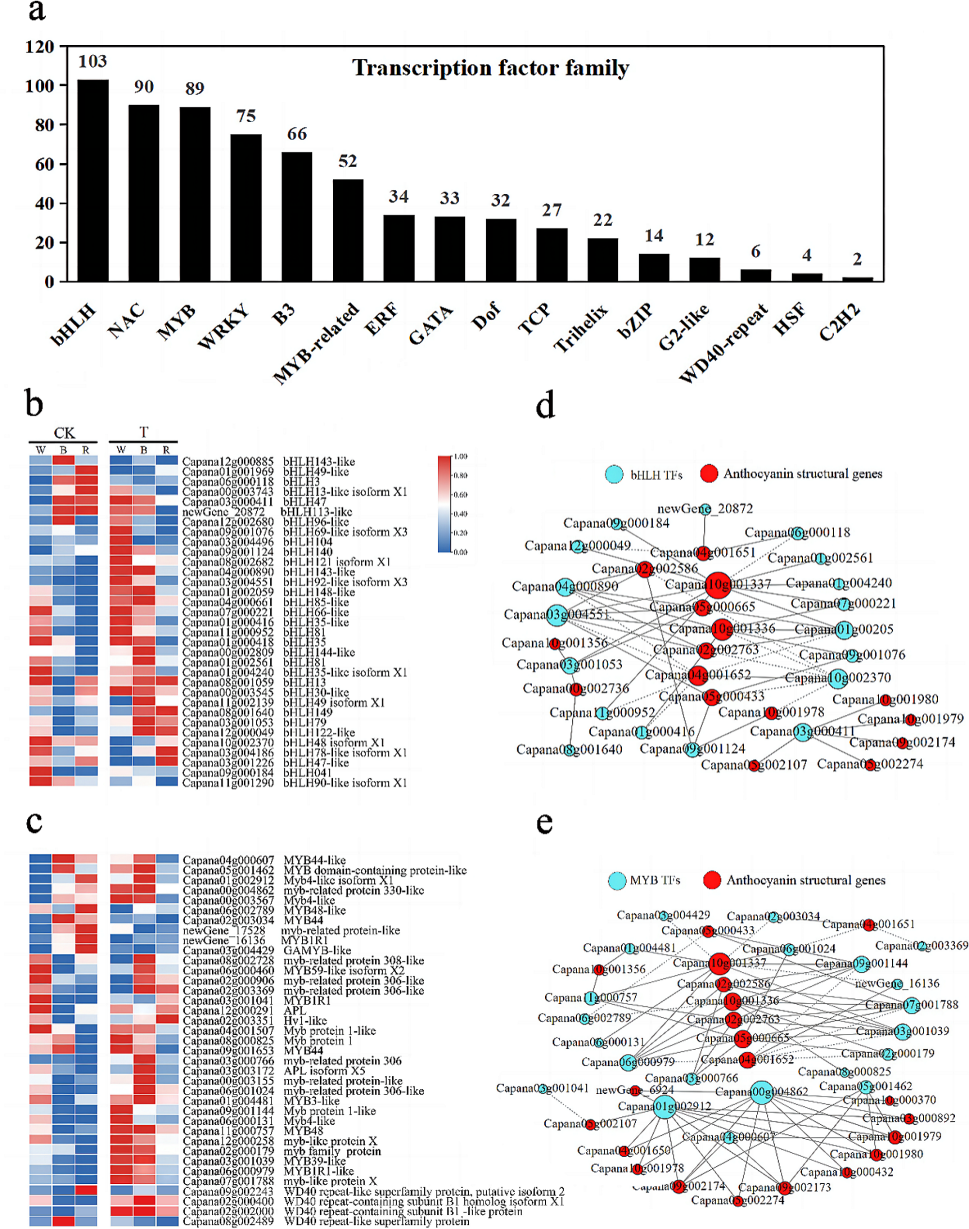
Analysis of transcription factors associated with the anthocyanin biosynthesis pathway. (**a**) Families of related transcription factors. (**b**) Heat map of bHLH transcription factors. (**c**) Heat map of MYB and WD40-repeat transcription factors. Gene expression was scaled using Z-scores of fragments per kilobase of exon per million fragments mapped (FPKM) for mean values of three biological replicates in heatmaps. W, White light; B, Blue light; R, Red light. CK indicates normal photoperiod irradiation; T indicates continuous photoperiod irradiation. (**d**) Correlation network analysis of anthocyanin structural genes with anthocyanin components, MYB TFs. (**e**) Correlation network analysis of anthocyanin structural genes with anthocyanin components, bHLH TFs. Only Pearson correlation coefficients (PCC) ≥ 0.80 or ≤ -0.80 are shown. solid line, positive correlation between genes; dashed line, negative correlation between genes

The anthocyanin synthesis pathway is regulated primarily by four transcription factor families, namely MYB, bHLH, WD40-repeat, and bZIP. In our study, a total of 16 transcription factor families were identified in pepper leaves under different light treatments, among which the proportions of MYB, bHLH, WD40-repeat, and bZIP were 13.4%, 15.6%, 0.9%, and 2.1%, respectively. (Fig. 7a, Table S6). The DEGs in the MYB, bHLH, and WD40-repeat transcription factor families were further analyzed, with a fragments per kilobase of transcript per million mapped reads (FPKM) value > 5 as the screening condition. The results showed that the levels of DEGs in the same transcription factor were not identical under different light treatments (Fig. 7b–c). Compared with the normal light treatment, the expression of transcription factors in the continuous light treatment increased; MYB1R1-like (Capana06g000979), MYB48 (Capana11g000757), MYB4-like isoform X1 (Capana01g002912), bHLH143-like (Capana04g000890), and bHLH92-like (Capana03g004551) were significantly upregulated in TW and TB, indicating that the biosynthesis of anthocyanin required higher transcriptional activity. There was no significant difference in the expression of the WD40-repeat protein between treatment groups. At the same time, we also found that the transcription factor Dof zinc finger protein DOF5.5 (*CDF1*), which regulates anthocyanin synthesis and exhibits highly dynamic changes in response to light (Table S7, Supplementary Fig. 2), showed significantly increased gene expression in the continuous light treatments compared with the normal light treatments, and gene expression was higher in the TB and TW treatments than in the TR treatment.

Further analysis of correlations between the structural genes in the anthocyanin biosynthesis pathway and transcription factors was performed (Fig. 7d–e). The results showed that MYB1R1-like, MYB48, bHLH143-like, and bHLH92-like isoform X3 were positively correlated with anthocyanin structural genes *F3H* (Capana02g002586), *FLS* (Capana10g001336 and Capana10g001337), and *DFR* (Capana02g002763 and Capana05g000665) and that MYB4-like isoform X1 (Capana01g002912) was positively correlated with *BZ1* (Capana10g001980), *FLS* (Capana09g002174), *F3’5’H* (Capana03g000892), and many other structural genes. These transcription factors may be involved in anthocyanin biosynthesis and the regulation of leaf color.

### qRT-PCR verification for RNA-Seq

**Fig. 8:**
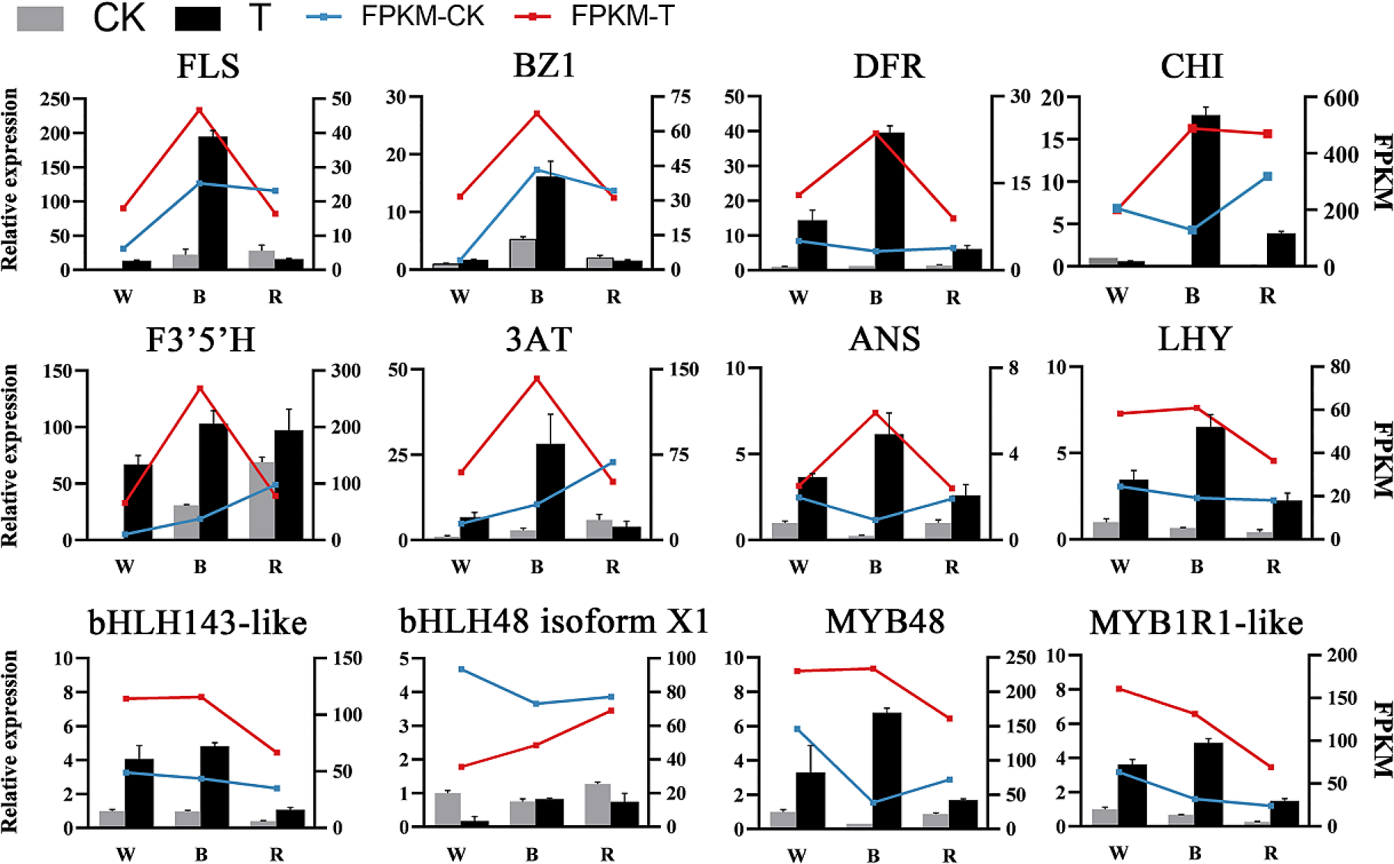
Validation and expression analysis of selected genes using qRT-PCR. The X-axis represents leaf samples from different light treatment groups; the left Y-axis represents relative gene expression levels (2^−ΔΔCt^) analyzed by qRT-PCR; the right Y-axis represents FPKM values obtained by RNA-seq. CK indicates normal photoperiod irradiation; T indicates continuous photoperiod irradiation. The error bars indicate the SDs of three biological replicates. W, White light; B, Blue light; R, Red light. *FLS*: Capana09g002174;*BZ1*: Capana10g001980; *DFR*: Capana05g000665; *CHI*: Capana00g002736; *F3’5’H*: Capana03g000892; *3AT*: Capana10g000432; *ANS*: Capana10g001356; *LHY*: Capana08g000974; *bHLH143-like*: Capana04g000890; *bHLH48 isoform X1*: Capana10g002370; *MYB48*: Capana11g000757; *MYB1R1-like*: Capana06g000979

To examine the results of RNA-Seq sequencing, we selected 12 DEGs for qRT-PCR experiments. By comparing the transcriptome abundance (FPKM) obtained from transcriptome sequencing, we found that the qRT-PCR results of the light-treatment-related genes were basically consistent with the trend of FPKM values, which proved that the RNA-Seq assay data in this study were reliable (Fig. 8).

## Discussion

Light has significant regulatory effects on plant growth and development, morphogenesis, physiological processes, material metabolites, and gene expression [40]. Horticultural plants perceive different light qualities through their photoreceptors, and this can cause changes in photosynthetic pigments [41]. It has been shown that eggplant [42], cucumber [43], and lily [44] seedlings have significantly more chlorophyll under red light than under blue light. Meng [9] showed that blue light was better than red light in promoting the synthesis and accumulation of anthocyanins in *Gerbera* plants. Hoffmann and Hern´andez et al. found that in pepper leaves and tomato seedlings, anthocyanin content increased with an increasing proportion of blue light [45, 46]. Strawberry fruits accumulated anthocyanin glycosides in response to different light qualities; the highest accumulation of anthocyanin glycosides was observed under blue light, while red light treatment caused a weaker induction of anthocyanin glycoside synthesis than blue or white light [15]. Anthocyanin content in European lingonberry fruit was significantly higher after a 24 h light treatment than a 12 h light treatment [47]. In our study, anthocyanin accumulation in pepper leaves was significantly higher after continuous blue light treatment than after red light treatment, while anthocyanin accumulation was also observed in pepper leaves under continuous white light (which contained blue light) treatment, indicating that anthocyanin biosynthesis in pepper leaves is affected by blue light and a long photoperiod.

The main types of anthocyanins present in nature are cornflower pigments, delphinium pigments, and geranium pigments, which are widely present in leaves, flowers, and fruits [48]. Among them, dihydroxy anthocyanidins are responsible for red color in fruits and leaves, and trihydroxy anthocyanidins are responsible for blue–purple color in plants [4, 49]. The highest cornflower pigment content (dihydroxy anthocyanidins) was found in the ‘Red Earth’ grape, while anthocyanidins in purple eggplant were mainly B-ring trihydroxy delphinidin 3-O-rutinoside and delphinidin 3-O-p-coumaroyl glucose [50, 51]. The seven anthocyanins that Luo et al. identified in purple shamrock were all derivatives of delphinidin [52]. In addition, delphinidin-3-O-glucoside and delphinidin-3-O-rutinoside were the predominant anthocyanins in blackcurrant [53]. Anthocyanins can be divided into light-induced and non-light-induced types. The major anthocyanins detected in purple leaves in this experiment included delphinidin-3-O-glucoside and delphinidin-3-O-rutinoside, both of which were significantly elevated after continuous blue and white light treatments, and anthocyanin content in the continuous blue light treatment group was significantly higher than in the other light treatment groups. This indicates that the main types of anthocyanins in pepper leaves were light-induced, continuous blue light irradiation regulated anthocyanin accumulation.

Differences in anthocyanin content may be due to the induction, by different light treatments, of gene expression related to the anthocyanin metabolic pathway. Two types of genes have been shown to influence anthocyanin biosynthesis: One type is a structural gene (an enzyme in the metabolic pathway that catalyzes anthocyanin biosynthesis), and the other type is a transcriptional gene that regulates structural genes [54–57]. Previous studies have shown that irradiation of cherries with blue or white–blue–green light increased the activity of *PAL* enzymes, resulting in higher anthocyanin content in the fruit [58]. In blueberry leaves, blue light and red/blue light promoted anthocyanin biosynthesis by inducing the expression of key structural genes and participating in the accumulation of metabolites in the anthocyanin synthesis pathway [59]. Blue light can also promote the expression of MrMYB1 and the anthocyanin synthesis-related structural genes *MrCHI*, *MrF3H*, *MrF3’H*, *MrDFR1*, *MrDFR2*, and *MrANS* in bayberry fruit, thus promoting anthocyanin synthesis [13]. By analyzing the expression of structural genes for anthocyanin synthesis in pepper leaves, we found that the expression of *PAL* (Capana05g002560), *F3H* (Capana02g002586), *DFR* (Capana05g000665), *BZ1* (Capana10g001980), and *ANS* (Capana10g001356) were significantly higher. In addition, *F3’5’H* is a crucial enzyme for the synthesis of delphinidin in the anthocyanin biosynthesis pathway, and the abundance of the *F3’5’H* gene at the transcriptional level can affect the composition of blue anthocyanin [60]. Yukihisa et al. [61] successfully obtained a new blue–purple rose with delphinidin accumulation by transferring *F3’5’H* into roses and overexpressing it. In the present experiment, delphinidin was dominant in purple pepper leaves, and the expression of *F3’5’H* (Capana03g000892) was positively proportional to anthocyanin content under continuous blue light and continuous white light irradiation. Therefore, we speculate that light duration and blue light irradiation induced high expression of *F3’5’H*, causing pepper leaves to accumulate delphinidin-3-O-rutinoside and delphinidin-3-O-glucoside in large amounts and to change from green to purple.

Transcription factors regulate the structural genes of anthocyanin biosynthesis and promote anthocyanin accumulation in plants [31]. Previous studies have found that *MdMYB1* and *MdMYBA* are key regulatory factors in anthocyanin accumulation and fruit color in apples in response to light induction, and MYB is thought to be related to the regulation of the blue light response [62, 63]. The MYB and bHLH transcription factors in peanut seed coats regulate the structural genes *DFR* and *ANS* in the anthocyanin biosynthesis pathway [64]. The structural genes *F3H* and *F3’H* in the anthocyanin synthesis pathway in ‘RP’ *Lobelia* are consistent with transcriptional level expression trends in *MYB* and *bHLH* [65]. *PhJAF13* and *PhAN1* are the main *bHLH-like* regulators of anthocyanin biosynthesis in *Petunia*; they both interact with *PhAN2* to activate *DFR* expression and *PhAN1* can also directly activate *DFR* expression [66]. Qiu et al. [67] found that the *SlAN1* gene in tomato overexpression plants was always co-expressed with *SlF3’5’H* and *SlDFR*, thus enhancing anthocyanin accumulation in the tomato pericarp. In the present study, *MYB1R1-like*, *MYB48*, *MYB4-like isoform X1*, *bHLH143-like*, and *bHLH92-like isoform X3* had significantly elevated expression under continuous blue light. Correlation network analysis revealed a strong, positive correlation between these transcription factors and structural anthocyanin synthesis genes *F3H*, *DFR*, *ANS*, and *F3’5’H*. These results showed that transcription factors caused the accumulation of anthocyanins by regulating the expression of anthocyanin synthesis structural genes. However, whether these transcription factors regulate the expression of anthocyanin structural genes by mediating long-day and blue light responses remains to be verified. At present, a large number of studies have confirmed that the WD40 gene *TTG1* regulates the anthocyanin biosynthesis pathway with bHLH and MYB transcription complexes [30, 67]. The expression of *StWD40*, *StAN1*, and *StbHLH* was significantly upregulated in red and purple fleshy tubers of potato [68]. In this study, although there was no significant difference in the levels of the WD40-repeat protein between the groups, whether it forms a complex with MYB and bHLH proteins requires further verification.

The anthocyanin biosynthetic pathway has numerous steps and is also tightly regulated by circadian rhythms, forming a complex secondary metabolic network [17]. Three components of the central oscillator of the circadian system, *LHY*, *CCA1*, and *TOC1*, constitute the core negative feedback loop that regulates circadian rhythms in Arabidopsis, rice, and other plants [69–71]. *COP1*, located downstream of the photoreceptor, acts as the main ligase for photomorphogenesis [72], photoperiodic growth [73], and anthocyanin biosynthesis [74] in plants induced by light. Li showed that *COP1* proteins are involved in light-induced anthocyanin biosynthesis by binding to the photoreceptor [74]. *MdCOP1* interacts with *MdMYB1* to regulate apple fruit color, and *MdMYB1* is degraded by *MdCOP1* in dark conditions [75]. Maier and Hoecker discovered that the *COP1/SPA1* complex is an essential suppressor of light signaling, and its mutation leads to excessive accumulation of anthocyanins under normal and high-intensity light [76]. Jiang et al. [77] found that blue light induced a *CRY1/CRY2-COP1* interaction in eggplant and promoted the binding of *SmMYB* to promoters of anthocyanin synthesis structural genes, such as *SmCHS* and *SmDFR*. In this study, continuous blue light and continuous white light irradiation activated anthocyanin biosynthesis in pepper leaves, and we found that *LHY* and *COP1* were significantly expressed under continuous blue light irradiation. Our analysis showed that *LHY* and *COP1* were positively correlated with several anthocyanin structural genes, including *CHS*, *CHI*, *F3H*, *F3’5’H*, *DFR*, and *BZI*. Therefore, we concluded that due to the change of light/dark cycle under continuous blue light treatment, the disappearance of dark conditions in the circadian rhythm system of pepper plants led to continuous expression of *LHY*, and an end to the inhibition of *LHY* by *TOC1.* At the same time, continuous light caused *COP1* to lose its function of degrading anthocyanin synthesis gene expression and to participate in different ways in photo-induced anthocyanin biosynthesis in pepper leaves. This agrees with previous studies [78, 79]. In conclusion, blue light and a prolonged photoperiod promoted anthocyanin formation by means that are not specific.

## Conclusion

**Fig. 9:**
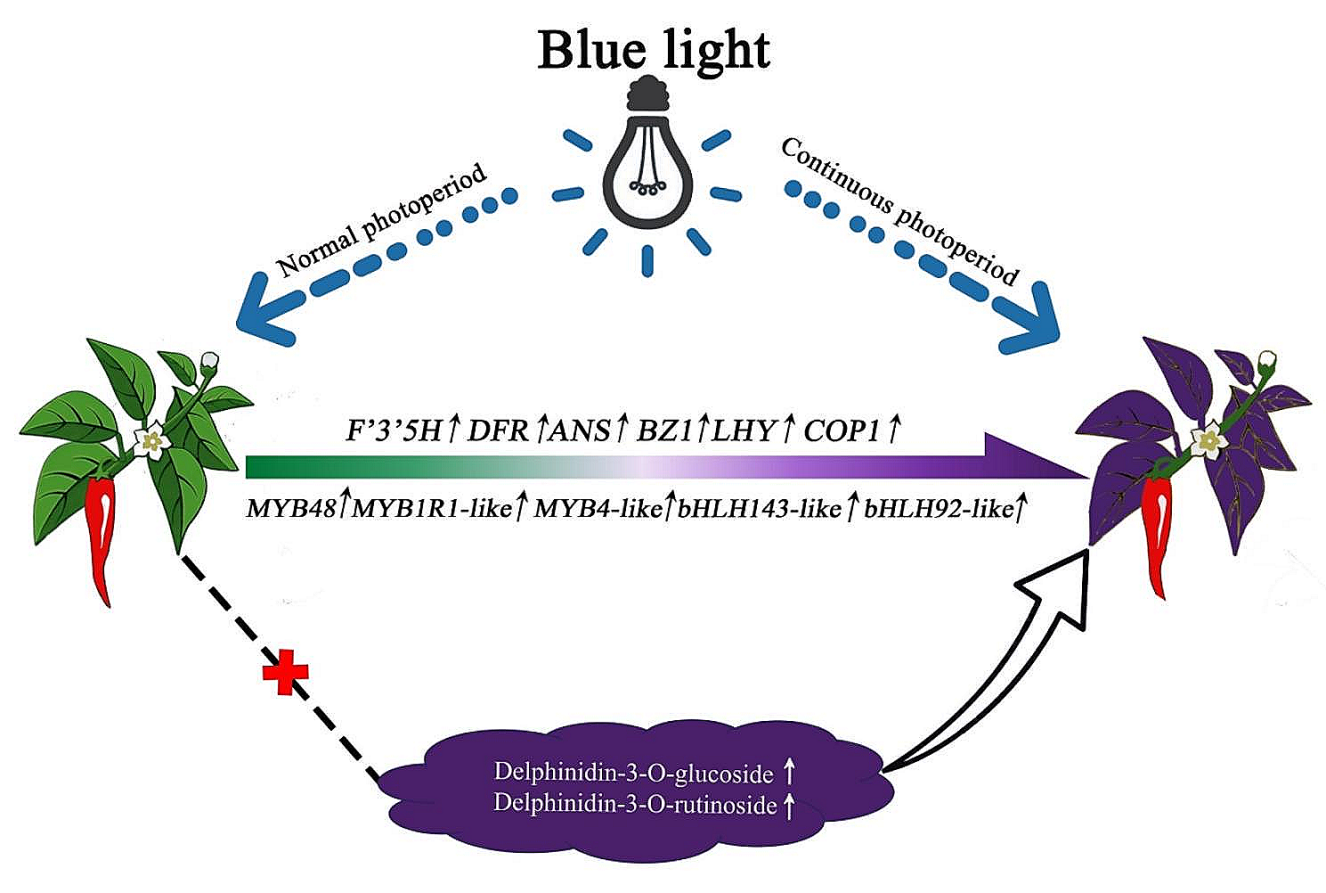
Model diagram of light-induced anthocyanin biosynthesis

Transcriptional analysis revealed 16,243 differentially expressed genes in pepper leaves, including 4,544, 7,936, and 5,023 genes in the TW/CW, TB/CB, and TR/CR treatments, respectively. Identification and functional analysis of DEGs showed that continuous blue light irradiation significantly increased the expression of *LHY*, *COP1*, *F3H*, *F3’5’H*, and *BZ1* genes in the circadian rhythm-plant pathway and anthocyanin metabolism pathway, which affected the expression of genes related to the MYB and bHLH transcription factor families. Correlation analysis showed that *LHY*, *COP1*, *MYB1R1-like*, *MYB48*, *MYB4-like isoform X1*, *bHLH92-like isoform X3*, and *bHLH143-like* genes were positively correlated with the structural genes in the anthocyanin synthesis pathway. Metabolic analysis showed that delphinidin-3-O-glucoside and delphinidin-3-O-rutinoside accumulated significantly in pepper leaves under continuous blue light irradiation. We observed the regulatory mode of light-induced anthocyanin biosynthesis in pepper leaves (Fig. 9); this mode showed that blue light and 24 h irradiation jointly activated structural genes and transcription factors in the anthocyanin biosynthesis pathway of pepper leaves. This study presents a mechanistic basis for anthocyanin anabolic metabolism induced by light quality and photoperiod in pepper leaves and provides a reference for screening plant anthocyanin biosynthesis as regulated by a reasonable light ratio.

## Materials and methods

### Plant materials and treatment

Seeds were soaked in warm broth, placed in an artificial climate chamber for germination, and then sown in 50-hole cavity trays where they received 16 h of light at 28 °C and 8 h of darkness at 20 °C. Seedlings received normal water and fertilizer management. When they had four leaves and one heart, seedlings were transplanted to 32-hole seedling trays and then placed under white (W), blue (B), and red (R) light-emitting diodes (LEDs) for 15 d. Photoperiodic setting were as follows: 14 h light and 10 h dark for the normal photoperiodic irradiation group (CK) and 24 h light and 0 h dark for the continuous photoperiodic irradiation group (T), with a diurnal temperature of 28 °C (day) and 20 °C (night) and humidity of 65 ± 5%. There were 40 seedlings in each treatment group and three replications. The photosynthetic photon flux density (PPFD) was maintained at 200 ± 5 µmol/m^2^·s in each treatment. The phenotypes of normal white light treatment ( CW ), normal blue light treatment ( CB ), normal red light treatment ( CR ), continuous white light treatment ( TW ), continuous blue light treatment ( TB ), and continuous red light treatment ( TR ) were observed and photographed before and after treatment, and functional pepper leaves were sampled for the subsequent experiments.

### Determination of phenotypic indicators of pepper plants

Whole pepper plants were sampled separately after light quality treatment. Plant height was measured with a ruler, stem thickness was measured with vernier calipers (accuracy 0.001 m), roots were washed with water and blotted with paper, and the fresh weight of the whole plant was obtained on a balance with an accuracy of one-tenth of 1%. The fresh samples were oven dried at 60 ℃ for two to three days and then the dry weight of the whole plant was determined. Each treatment was repeated three times, and the average value was taken.

### Determination of pigment indicators in pepper leaves

Leaf color differences for the CW, CB, CR, TW, TB, and TR phenotypes were measured using a spectrophotometer (Ts7600, Shenzhen 3nh Technology Co., Ltd., Shenzhen, China). Six leaves from the same location on three plants were selected from each treatment group for measurement. The color difference parameters △L (light and dark), △a (red–green), and △b (yellow–blue) were obtained, and the total chromaticity value △E was calculated. Functional leaves with significant phenotypic changes were selected, and three plants were chosen from each treatment group, with three replications. The absorbance values of the sample extracts at A663, A645, and A470 nm were determined using a multifunctional enzyme marker (TECAN/ SPARK), and chlorophyll a, chlorophyll b, and carotenoid contents were calculated after 24 h maceration with 80% acetone (until the material whitened), following the method of Arnon [80]. Total anthocyanin glycosides were extracted via the methanol-hydrochloric acid method. Samples (0.5 g) were ground with liquid nitrogen and incubated in 5 ml of methanol containing 0.1% (v/v) hydrochloric acid at room temperature and protected from light overnight. The absorbance of the sample extracts at 520 and 700 nm was measured using a multifunctional enzyme marker (TECAN/ SPARK) and the anthocyanin content of the samples was measured by pH difference [81].

### RNA extraction, library preparation, and sequencing

Total RNA was extracted from tissues using TRI reagent (Sigma Life Science, USA) according to the manufacturer’s instructions, checked for quality by ribonuclease-free agarose gel electrophoresis to avoid possible degradation and contamination, and validated using an Agilent 2100 Bioanalyzer (Agilent Technologies, Santa Clara, CA, USA). Poly(A) mRNA was then isolated using oligo-dT beads (Qiagen, Germany) and later fragmented into short fragments by adding a fragmentation buffer. First-strand cDNA was synthesized via reverse transcription with random hexamer primers using mRNA as a template, and second-strand cDNA was synthesized by adding buffer, RNase H, and DNA polymerase I. The cDNA fragments were purified with a QIA rapid PCR extraction kit and then washed with EB buffer for end-repair poly(a) addition and ligated to sequencing adapters. After agarose gel electrophoresis and extraction of cDNA from the gel, cDNA fragments were purified and enriched by PCR to construct the final cDNA library, which was sequenced using paired-end technology on the Illumina sequencing platform (Illumina-nova6000). Three biological replicates were performed for each strain, resulting in 18 differential gene expression (DGE) libraries.

### Transcriptome analysis

After sequencing, raw reads were filtered using Perl software to remove low-quality sequences (more than 50% of a sequence with a base mass below 20 or a sequence containing more than 5% N-base (unknown) reads) and reads containing adapter sequences. Clean reads were mapped to the pepper reference genome using TopHat2, allowing for at most one mismatch [82]. All successfully mapped transcripts were identified using the R package “edgeR” against DEGs, and expression levels were calculated for each gene and normalized to FPKM. The false discovery rate (FDR) was used to determine thresholds for *p*-values across multiple experiments. Transcripts with an FDR < 0.05 were considered significant and served as significant cut-offs for gene expression differences and for GO and KEGG enrichment analysis. GO terms with corrected *P-values* < 0.05 and KEGG pathways with *P-values* < 0.05 were considered significantly enriched for differentially expressed genes. The GO (http://www.geneontology.org/) functional database and KEGG (https://www.genome.jp/kegg/) pathway database were used to perform enrichment analysis on the differential gene set.

### Metabolite extraction

Pepper leaf tissue was freeze-dried and ground into powder with a grinder (30 Hz, 1.5 min), and 50 mg powder was extracted in 0.5 mL methanol/water/hydrochloric acid (500:500:1, v/v/v). Then, the extract was vortexed for 5 min, ultrasonicated for 5 min, and centrifuged at 12,000 g for 3 min at 4 °C. The residue was re-extracted by repeating the above steps under the same conditions. The supernatants were collected and filtrated through a membrane filter (0.22 μm, Anpel) before LC-MS/MS analysis [83].

### HPLC-MS/MS analysis

LC-MS/MS analysis was performed with an ExionLC™ AD system (SCIEX) from New Genetics Corporation (Beijing, China) coupled to a QTRAP® 6500 + mass spectrometer (SCIEX). Samples were injected into an Xselect HSS T3 (2.1 × 150 mm, 2.5 μm) using a 20 min linear gradient with a flow rate of 0.4 mL/min for the positive/negative polarity mode. The eluents were eluent A (0.1% formic acid in water) and eluent B (0.1% formic acid in acetonitrile) [84]. The solvent gradients were set as follows: 2% B, 2 min; 2–100% B, 15.0 min; 100% B, 17.0 min; 100–2% B, 17.1 min; and 2% B, 20 min. The QTRAP® 6500 + mass spectrometer was operated in positive polarity mode with a twilight gas of 35 psi, medium collision gas, ion spray voltage of 5500 V, and temperature of 550 °C. The QTRAP® 6500 + mass spectrometer was operated in negative polarity mode with a twilight gas of 35 psi, dielectric collision gas, ion spray voltage of − 4500 V, temperature of 550 °C, ion source gas 1:60, and ion source gas 2:60.

### Quantitative real-time PCR

Differentially expressed genes identified by transcriptome analysis were detected by quantitative real-time PCR. qRT-PCR was performed by Taylor et al. [85], using Actin as the internal reference gene, and three replicates of each sample were performed using the Vazyme fluorescence quantification kit (ChamQTM SYBR® qPCR Master Mix, Jiangsu, China) qRT-PCR validation was performed. The relative expression levels of genes were normalized by the 2^−ΔΔCt^ method [86]. qRT-PCR primer sequences are shown in Table S8.

### Electronic supplementary material

Below is the link to the electronic supplementary material.

Supplementary Material 1

Supplementary Material 2

Supplementary Material 3

Supplementary Material 4

Supplementary Material 5

Supplementary Material 6

Supplementary Material 7

Supplementary Material 8

Supplementary Material 9

Supplementary Material 10

Supplementary Material 11

Supplementary Material 12

## Data Availability

The data presented in this study are available on request from the corresponding author. RNA-Seq data generated in this study are available from the SRA-Archive (http://www.ncbi.nlm.nih.gov/sra) with accession number PRJNA980944.

## Abbreviations

DEGs: Differentially expressed genes
CW: Normal white light irradiation
CB: Normal blue light irradiation
CR: Normal red light irradiation
TW: Continuous white light irradiation
TB: Continuous blue light irradiation
TR: Continuous red light irradiation
*PAL*: phenylalanin ammonialyase
*4CL*: 4-coumarate:CoA ligase
*CHS*: chalcone synthase
*CHI*: chalcone isomerase
*F3H*: flavonoid 3-hydroxylase
*FLS*: flavonol synthase
*F3’H*: flavonoid3’-hydroxylase
*F3’5’H*: flavonoid 3’5’-hydroxylase
*DFR*: dihydroflavonol 4-reductase
*ANS*: anthocyanidin synthase
*BZ1*: anthocyanidin 3-O-glucosyltransferase
*3AT*: anthocyanidin 3-0-glucoside 6"O-acyltransferase
*LHY*: MYB-related transcription factor LHY
*COP1*: E3 ubiquitin-protein ligase RFWD2
*SPA1*: protein suppressor of PHYA-105 1
