# Supplementary figures and images for: Integrated transcriptome and metabolome analysis reveals anthocyanin biosynthesis mechanisms in pepper (Capsicum annuum L.) leaves under continuous blue light irradiation
Source: BMC Plant Biol. 2024 Mar 23;24:210. doi: 10.1186/s12870-024-04888-x (PMC10960449; doi:10.1186/s12870-024-04888-x)

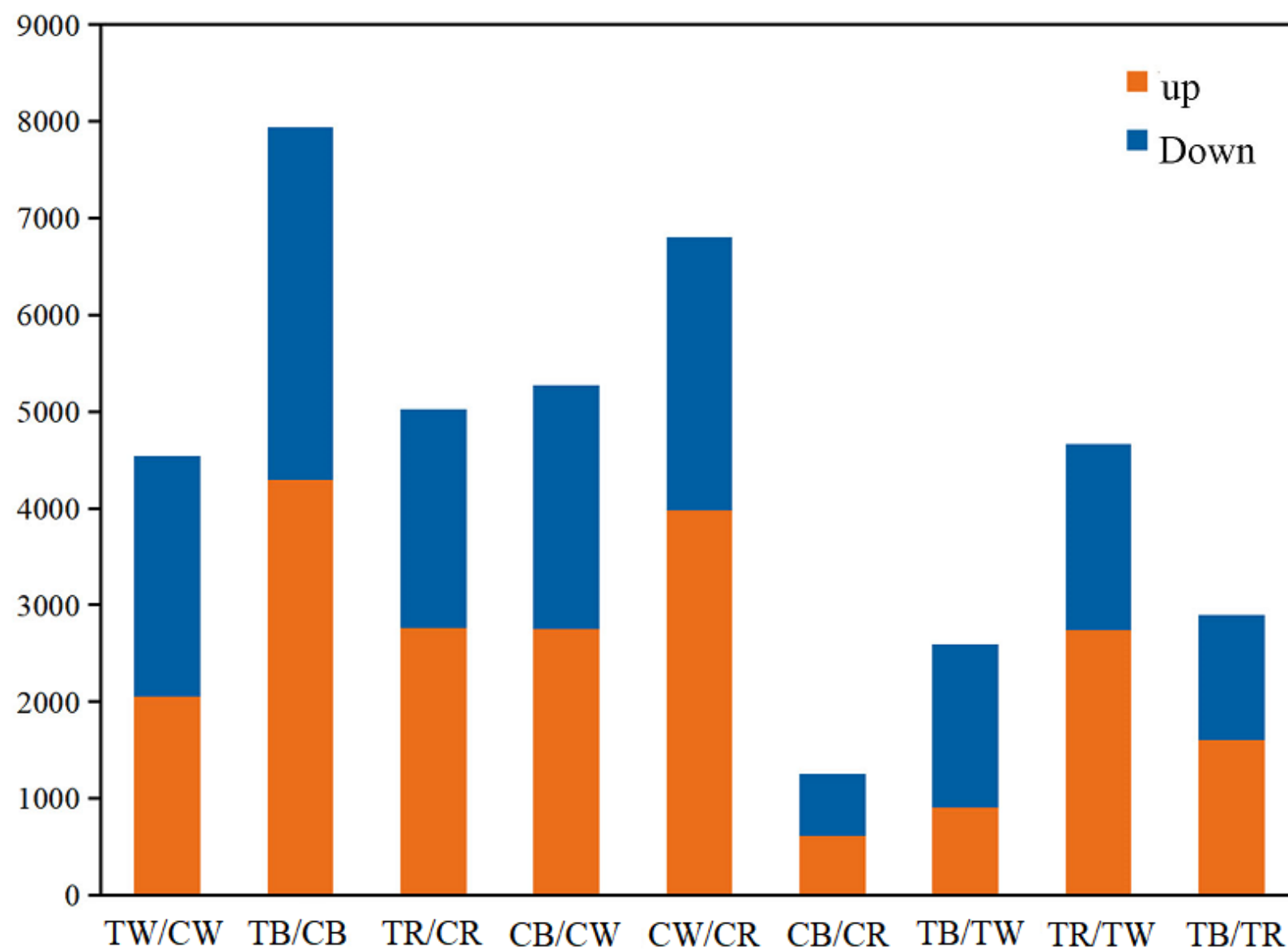

Figure S1: DEGs in each comparison group.

Supplement: Supplementary file 11 — Supplementary Material 11 [file 12870_2024_4888_MOESM11_ESM.pdf]

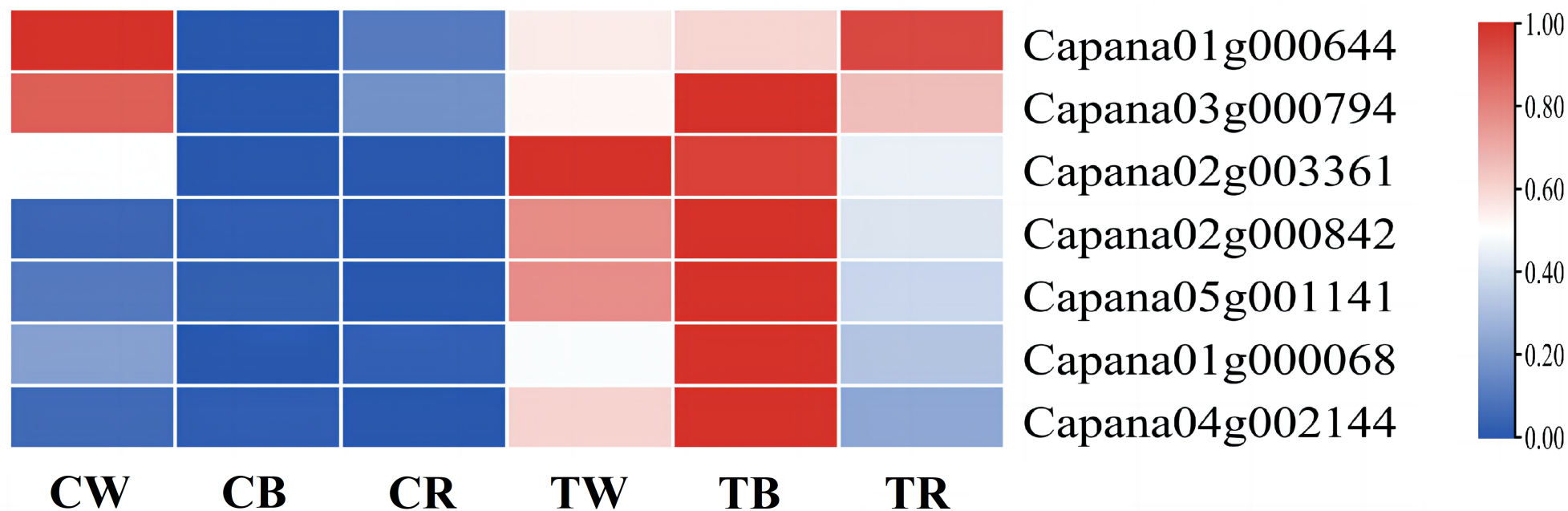

Figure S2: Heat map of DOF transcription factors.

Supplement: Supplementary file 12 — Supplementary Material 12 [file 12870_2024_4888_MOESM12_ESM.pdf]
